# The Efficacy and Safety of Traditional Chinese Medicine (Jiang Zhi Granule) for Nonalcoholic Fatty Liver: A Multicenter, Randomized, Placebo-Controlled Study

**DOI:** 10.1155/2013/965723

**Published:** 2013-12-04

**Authors:** Jielu Pan, Miao Wang, Haiyan Song, Lin Wang, Guang Ji

**Affiliations:** ^1^Institute of Digestive Disease, Longhua Hospital, Shanghai University of Traditional Chinese Medicine, Shanghai 200032, China; ^2^Department of Social Sciences, Shanghai University of Traditional Chinese Medicine, Shanghai 201203, China; ^3^E-Institute of Shanghai Municipal Education Commission, Shanghai University of Traditional Chinese Medicine, Shanghai 201203, China

## Abstract

*Objective*. To evaluate the efficacy and safety of Jiang Zhi Granule (JZG), a Chinese herbal formula, in patients with nonalcoholic fatty liver (NAFL). *Methods*. A multicenter, randomized, double-blind, placebo-controlled, parallel clinical trial was conducted for 24 weeks in 224 patients with NAFL at 6 university-affiliated hospitals. Patients were randomized 1 : 1 to receive JZG and placebo, respectively. Primary outcome was the change of liver to spleen ratio (L/S ratio) over computed tomography (CT). Secondary outcomes included body mass index (BMI), serum triglyceride (TG), and total cholesterol (TC) levels. *Results*. Of all the 224 eligible patients, 221 patients were analyzed in the full analysis set (FAS), 205 in the per protocol set (PPS), and 3 patients were withdrawn prematurely. For FAS, JZG significantly increased L/S ratio from 0.74 ± 0.21 to 0.99 ± 0.24 compared to that from 0.79 ± 0.18 to 0.85 ± 0.27 in placebo group (*P* = 0.0011). For PPS, it showed an increase of 0.26 ± 0.23 of L/S ratio in the patients on JZG versus 0.07 ± 0.22 in those on placebo (*P* = 0.0003). Superiority of JZG over placebo was also observed with greater reduction in BMI (*P* < 0.05) in both FAS and PPS. No observable difference in decrease of serum TC and TG was recorded (*P* > 0.05). There were no serious adverse events (AEs) in the study process and safety indices were normal in both groups. *Conclusions*. The Chinese herbal formula JZG was found to be superior to placebo in increasing L/S ratio and reducing BMI in NAFL patients. It was also well tolerated in patients and might be a safe and effective medicine for NAFL.

## 1. Introduction

Fatty liver disease is a hepatic manifestation, mainly featured with hepatocytic steatosis, without excess alcohol intake history; the condition is called nonalcoholic fatty liver disease (NAFLD) [1, 2]. The clinicopathological spectrum of NAFLD ranges from simple steatosis (nonalcoholic fatty liver, NAFL) to nonalcoholic steatohepatitis (NASH), even fibrosis and cirrhosis [3]. NAFLD is pervasive worldwide with NAFL as the initial feature. NAFL is common in the obese, which has the potential to progress to NASH, even fibrosis and cirrhosis, and is also correlated with type 2 diabetes mellitus, dyslipidemia, and hypertension [4].

The mechanisms under fatty liver are still elusive; insulin resistance, which is the most widely accepted pathogenesis, is proposed to be associated with hepatic steatosis [1, 5]. Up to now, there is no confirmed effective conventional therapy for NAFL, except for lifestyle modification and control of associated metabolic disorders. Nevertheless, new treatments are still in urgent need from basic research to bedside [6, 7].

Jiang Zhi Granule (JZG) is a clinically-used herbal formula designed for treating patients with fatty liver disease applyed in Longhua Hospital for more than a decade. It processes favorable treatment effects which have been testified by substantial laboratory researches [8, 9]. Previous studies on JZG have indicated that it had an antisteatotic effect on both cell lines and animals [8, 10].

The primary aim of this study was to compare the efficacy and safety of JZG in patients of NAFL with those of placebo. It is the first multicenter, double-blind, randomized controlled study of JZG for NAFL. The trial was approved by State Food and Drug Administration (SFDA) with certification (no. 2008L11181) and the Ethics Committee of Shanghai University of Traditional Chinese Medicine (no. [2010]-353).

## 2. Patients and Methods

### 2.1. Patients and Settings

The study was carried out on 245 patients with NAFL, randomized in 6 hospitals. Patients were considered for the trial if they had an identified diagnosis of NAFL and performed for 24 weeks of recruitment. The diagnosis criterion was based on the guidelines for the diagnosis and treatment of nonalcoholic fatty liver [11]. Other inclusion criteria were (1) age 18–75; (2) alanine aminotransferase (ALT) level within normal range or slightly elevate but less than 1.5 times as the upper limit; (3) blood glucose ≤7.0 mmol/L; (4) without taking any other medicine for NAFL treatment in recent month. All patients were given written informed consent before recruitment.

Patients were meant to be excluded in the following circumstances. (1) Positive testing for hepatotropic viruses, hepatitis A, hepatitis B, hepatitis C, hepatitis D, and hepatitis E, and drug-induced liver disease or autoimmunity liver disease also included; (2) with a history of daily alcohol consumption ≥20 g for a period longer than 2 years at any time in the past 10 years; (3) combined with severe primary disease in heart, brain, kidney, endocrine, blood, metabolism, and gastrointestinal tract or suffered from mental disease; presence of other major diseases including type 1 diabetes; (4) serum creatinine (Cr) value exceeding upper limit; (5) currently taking medicine to lose weight or keep fit; (6) planned to be pregnant and in lactation or in contraception with other medicine; (7) patients with allergies, multidrug sensitivity, and hypersensitivity of test drug or its compositions; (8) seriously ill and could not give exact evaluation of efficacy and safety of the test drug; (9) unwilling to cooperate; (10) presumed to have taken other drugs forbidden in the trial.

### 2.2. Methods

#### 2.2.1. Trial Design

The study was designed as a multicenter, randomized, paralleled, double-blinded, placebo-controlled clinical trial. Protocol and patient consent forms were designed by the coordinating center and reviewed by all participating centers. The protocol conformed to ethical guidelines of the 2000 Declaration of Helsinki and Chinese Good Clinical Practice (CGCP), based on the Consolidated Standards of Reporting Trials (CONSORT) statement [12] and was approved by the Ethics Committee of Shanghai University of Traditional Chinese Medicine. The trial was conducted simultaneously at 6 centers as follows: Hubei Provincial Hospital of TCM, Yunnan Provincial Hospital of TCM, Liaoning Provincial Hospital of TCM, Ruikang Hospital Affiliated to Guangxi University of Chinese Medicine, The First Hospital of Human University of Chinese Medicine, and Affiliated Hospital of Hunan Academy of Chinese Medicine. Eligible subjects were randomly assigned to one of the two groups, the JZG group and the placebo group. All the groups then underwent a 24-week treatment period. Four visits in total were scheduled for each subject at baseline, week 4, week 12, and week 24. Patients were applied with related measurements at every follow-up visit. Moreover, urine pregnancy tests were performed for female participants of child-bearing potential at each visit.

According to the demands of SFDA, while considering 20% for drop-off and allocated proportion of the 2 groups (1 : 1), we determined 120 patients for each group and 40 for every participating center. The randomization was stratified and allocation sequences were based on random digits table generated by SAS 8.1 software. Patients and investigators were double-blinded to treatment throughout the duration of the trial. All data were imputed, then blocked, and managed by the statistical department, and no revision would be allowed to be performed on the data.

#### 2.2.2. Treatment Regiment

As reported previously, JZG is composed of Jiaogulan (*Herba Gynostemmatis*), Danshen (*Radix Salviae Miltiorrhizae*), Huzhang (*Rhizoma Polygoni Cuspidati*), Yinchen (*Herba Artemisiae scopariae*), and Heye (*Folium Nelumbinis) *[10]. The placebo was made of starch and sugar to achieve comparable color, outlook, and taste to JZG. Both regiments were entrusted from Jiangsu Shenhua Pharmaceutical Co., Ltd. (Lot no. 20100218, 5 g per sachet) to be produced. The regiments were given three sachets per day for each patient. No significant changes were made to the protocol or prespecified outcomes after trial commencement. Quality control is provided. The regiments were packed in sealed opaque aluminium sachets; only the treatment code and administrating instruction were outside the package to ensure successful blinding of patients.

#### 2.2.3. Outcome Assessments

Liver biopsy is the gold standard for diagnosing fatty liver; because of the invasive operation and potential risks, it is not applicable in all patients. As a substitute, we chose an imaging method for this study. It was reported that the ratio of computerized tomography (CT) numbers for the liver and the spleen shows a good inverse correlation with the degree of steatosis shown on histology (*r* = −0.87) [13]. The primary outcome was the abdominal CT value ratio of the liver to spleen (L/S ratio) which was performed at baseline and at every follow-up. The main criteria for improving evaluation of hepatic steatosis can be followed as L/S ratio is less than or equal to 1. Mild degree has a ratio less than or equal to 1 but more than 0.7; moderate degree has a ratio less than or equal to 0.7 but more than 0.5; severe degree has a ratio less than or equal to 0.5 [14]. Second outcomes were body mass index (BMI), serum triglycerides (TG), and total cholesterol (TC).

Compliance was assessed with sachet counts. Patients with less than 80% treatment compliance or who missed a visit were withdrawn. Meanwhile, all the patients were provided with standard advice on diet and physical exercise at each follow-up visit by physicians and dieticians.

#### 2.2.4. Safety and Adverse Events Assessments

Clinical data containing heart rate, respiration, blood pressure electrocardiogram (ECG), and related symptoms were recorded at each visit. Patients underwent routine blood and urine tests including red cell count (RBC), white cell count (WBC), platelet count, and hemoglobin (HB). Patients were also demanded to detect ALT, aspartate aminotransferase (AST), blood urea nitrogen (BUN), Cr, and glucose at both entry and end of the trial. The occurrence of adverse events (AEs) was monitored and recorded at every follow-up for safety set (SS) analysis.

### 2.3. Statistical Analysis

The statistical significance was defined as two-sided *P* value of <0.05. Data was present as mean (standard deviation, SD), frequency, and percentages. Baseline differences between the groups were assessed with the use of Student's *t*-test for normally distributed continuous variables and non parametric Mann-Whitney *U* test for the nonnormally distributed. For categorical variables, chi-squared test or Fisher's exact test was used. Comparisons between placebo and JZG groups including the primary outcome and secondary outcomes were conducted according to the intention-to-treat (ITT) principle and are analyzed by both full analysis set (FAS) and per protocol set (PPS). The “FAS” includes all patients randomized to treatment who received at least one dose of the assigned treatment. The “PPS” excluded patients who lost to follow-up, withdrew early from the trial, had major deviations from the planned time schedule, failed to complete the trial medication, with low compliance, or did not attend the final visit. Safety analyses were conducted on the safety set (SS), which was defined as all subjects who took at least one dose of trial medication. Missing data were imputed via last observation carried forward (LOSF) method. Patient compliance was calculated as (1 − (*Va* − *Vo*)/*Va*) × 100%. *Va* is the number of sachets that a patient received; *Vo* is the number of sachets returned. The value of either <80% or >120% was considered as low compliance. For biochemical indices and safety assessments, Wilcoxon signed-rank tests and the Cochran Mantel-Haenszel (CMH) *χ* ^2^ tests were utilized. The statistical significance was set as two-tailed *P* value <0.05. The analysis was performed by SAS 8.1 (SAS Institute Inc., Cary, NC) and GraphPad Prism 5 (GraphPad Software, Inc., San Diego, USA).

## 3. Results

### 3.1. Participant Flow

**Figure 1:**
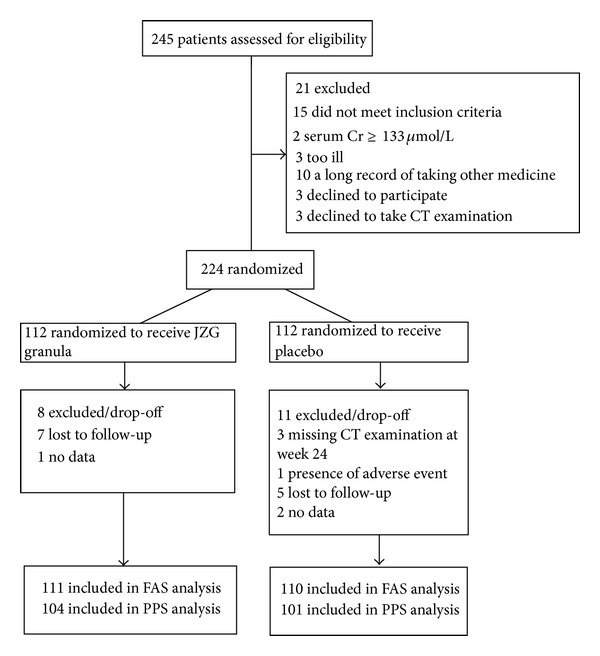
Patient flow diagram of the 2 trial groups.

The trial was conducted from March 1, 2010, to September 30, 2011. Patient screening, enrollment, and retention by treatment process were detailed in Figure 1. In total, 245 patients were recruited at 6 participating centers for primary screening. 224 patients participated in baseline eligibility screening for randomization; 21 patients were screened out due to the failure to meet inclusion standard. Eventually, 221 were included in FAS (111 in JZG group and 104 in placebo) and 205 in PPS (110 in JZG group and 101 in placebo). The total drop-off rate was 8.48% (9.82% and 7.14% for JZG and placebo groups, resp.).

### 3.2. Baseline Data

221 patients entered the trial (JZG group, male/female 94/17; placebo group, male/female 83/27). The baseline characteristics of the participants under FAS analysis were summarized in Table 1. The mean age of JZG group was 42.39 ± 11.55 years and the mean age of placebo group was 44.82 ± 11.41 years. Both L/S ratio and BMI were comparable between groups (*P* > 0.05). Other parameters (bodyweight, blood pressure, blood glucose, ALT, AST, BUN, Cr, etc.) were all well balanced (*P* > 0.05).

### 3.3. Primary Outcome

At week 12, the mean L/S ratio increased from 0.74 ± 0.21 to 0.87 ± 0.22 in JZG group compared to the ratio from 0.79 ± 0.18 to 0.82 ± 0.23 in placebo group under FAS, that was 0.13 ± 0.18 increase in JZG group versus 0.03 ± 0.18 in placebo group (*P* = 0.0003). For PPS, the mean L/S ratio increased from 0.74 ± 0.21 to 0.87 ± 0.22 on JZG compared to the ratio from 0.78 ± 0.18 to 0.82 ± 0.24 on placebo, which showed 0.13 ± 0.18 increase in JZG group versus 0.04 ± 0.18 in placebo group (*P* = 0.0015). At week 24, the mean L/S ratio increased from 0.74 ± 0.21 to 0.99 ± 0.24 in JZG group compared to the ratio from 0.79 ± 0.18 to 0.85 ± 0.27 in placebo group under FAS, that was 0.25 ± 0.23 increase in JZG group versus 0.06 ± 0.21 in placebo group (*P* = 0.0011). For PPS, the mean L/S ratio increased from 0.74 ± 0.21 to 0.87 ± 0.22 on JZG compared to 0.78 ± 0.18 to 0.82 ± 0.24 on placebo, which showed 0.13 ± 0.18 increase in JZG group versus 0.04 ± 0.18 in placebo group (*P* = 0.0015).  Table 2(a) reported the value of L/S ratio in both groups.

Furthermore, the difference in mean change from baseline between JZG and placebo was estimated using adjusted means together with the 95% CI from the ANCOVA model (Table 2(b)). Groups and centers were as factors and baseline was as covariance. FAS analysis showed that the difference value was −0.18 (95% CI, −0.24, −0.12). PPS analysis showed that the difference value was −0.18 (95% CI, −0.24, −0.12).

### 3.4. Secondary Outcomes

**Figure 2:**
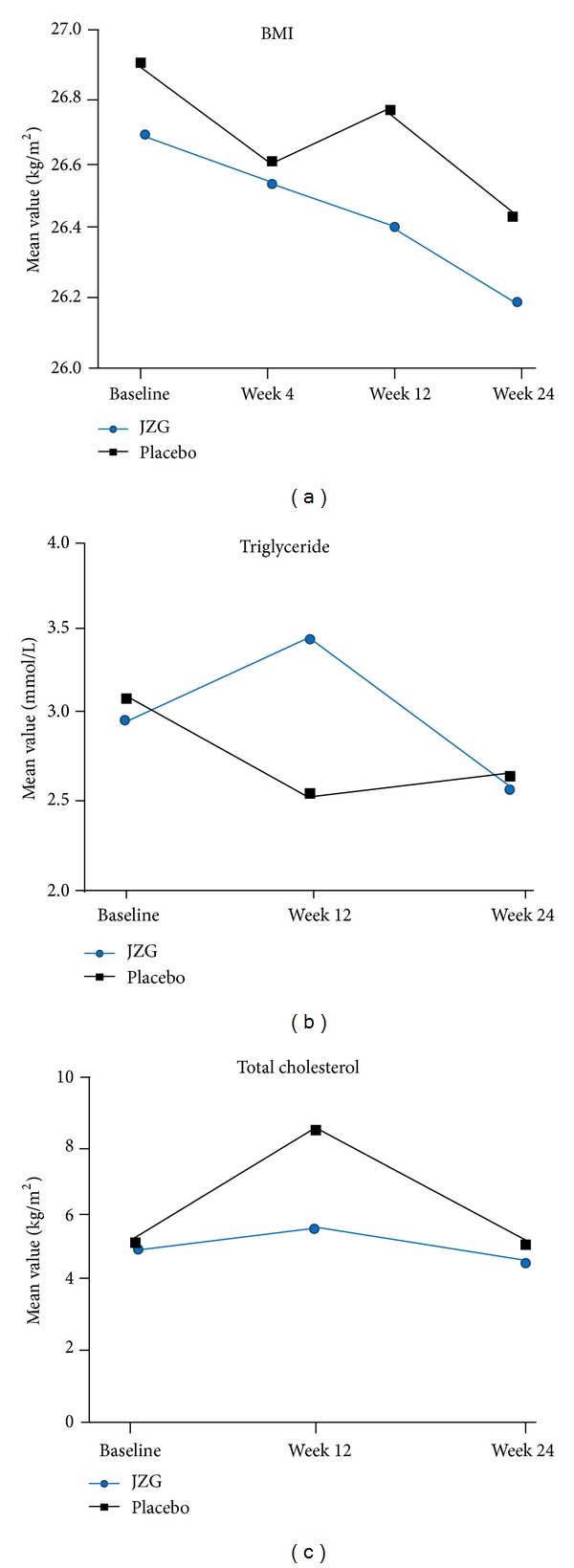
Changes in secondary outcomes (FAS) of the treatment. (a) BMI: body mass index. *P < 0.05 versus baseline. **P < 0.05 versus baseline; (b) serum TG: triglyceride; (c) serum TC: total cholesterol.

The changes in BMI, serum TG, and TC from baseline to week 4, week 12, and week 24 were illustrated in Figure 2. Significantly, reduction of BMI was demonstrated in both JZG and placebo groups. After the 24-week period, BMI was decreased from 26.69 ± 2.78 to 26.20 ± 2.87 in the JZG group and from 26.85 ± 3.34 to 26.38 ± 3.24 in the placebo group. At week 4 and week 12, there was no statistical difference observed from baseline or between groups. Reduction of serum TG and TC was observed at week 24 from baseline in both groups. Interestingly, week 12 witnessed a slight increase of serum TG on JZG and serum TC on both treatment.

### 3.5. Compliance Assessment

All patients were assessed with good compliance. Patients followed the direction of treatment strictly with an assessment value of 100%. Other combined medications not the patients took during the treatment were used for an identified diagnosis of disease and with allowance for trial protocol.

### 3.6. Safety and Adverse Events

The occurrence of AEs was monitored and recorded throughout the study, and no severe adverse events occurred during the process. There were 221 patients under SS analysis. 38 cases (of 30 patients) were reported with AEs (Table 3). Two cases (of 2 patients) were eventually determined as related to the study. Of all the AE cases, 19 cases (of 16 patients) occurred in JZG treatment and 19 cases (of 14 patients) in placebo intervention. The frequency of AE occurrence in JZG and placebo was 14.41% and 12.73%, respectively (Table 4). The study related to AEs was reported as 1 case with sloppy stool and the other case with exhaustion/dizziness. The 2 cases of AEs were all mild and transient and neither of the patients were withdrawn from the trail.

## 4. Discussion

The epidemic of NAFLD is prevailing in China; the morbidity has risen to 15–30% in some cities [15]. As the hepatic manifestation of metabolic syndrome, NAFLD is also a potential risk factor of type 2 diabetes and cardiovascular disease, which arouses a wide concern in various aspects. Since there are no specific drugs available in treating NAFLD, traditional Chinese medicine becomes one of the countable alternatives.

As more and more clinical trials were conducted in NAFLD, several consensuses were formed in the methodologies. NAFLD is composed of a spectrum of diseases including NAFL, NASH, fibrosis, and HCC; each stage has its specific methods of diagnosis, evaluation, and treatment. For the consideration of safety and quality control, the initial attempt of TCM application in NAFLD is mainly focusing on the early stage of the disease (NAFL). To perform fatty liver clinical trial, liver biopsy is the gold standard for diagnosing. However, due to the invasive operation and potential risks, it could not be applicable in all patients. Imaging techniques, including ultrasound (US), CT, and magnetic Resonance (MR), are now widely used. It was reported that CT attenuation values of the liver were strongly correlated with histological evidence of hepatic steatosis; the ratio of CT numbers for the liver and the spleen shows a good inverse correlation with the degree of steatosis shown on histology (*r* = −0.87) [13]. As a substitute, we chose an imaging method for this study.

According to TCM theory, fatty liver is corresponding to pathogens including dampness, heat, and stasis interacted in the liver with spleen deficiency as the basis. To target its pathomechanism, JZG has been applied in clinic for more than fifty years; it was designed to invigorate spleen, remove phlegm, eliminate dampness, activate blood flow, and clear heat. JZG is composed of Jiaogulan (*Herba Gynostemmatis*), Danshen (*Radix Salviae Miltiorrhizae*), Huzhang (*Rhizoma Polygoni Cuspidati*), Yinchen (*Herba Artemisiae scopariae*) and Heye (*Folium Nelumbinis*). Experimental studies have been conducted extensively for more than a decade and have focused on the phytochemistry and pharmacological action. Our previous studies have identified the active constitutes of JZG as tanshinone IIA, danshensu, salvianolic acid B, nuciferine, emodin, and chlorogenic acid through an ultraperformance liquid chromatography coupled with mass spectrometry [16]. The antisteatosis activity of each component was further testified in cell line, with FFA incubating for 24 hours; the HepG2 cells became “fatty,” while another 24-hour intervention with JZG component(s) witnessed the reducing lipid droplets in the cells [10]. A small clinical trial conducted by our lab demonstrated that combination of JZG with behavior intervention is superior to behavior intervention alone in lowering serum TG, increasing serum high density lipoprotein cholesterol, and reducing lipid content in the liver [17]. Further, in vitro and in vivo studies have confirmed that JZG alleviates hepatic steatosis through the inhibition of LXR alpha-SREBP1c pathway [8]. Animal studies using high fat diet-induced fatty liver rats demonstrated that JZG may have an effort on leptin pathway [18]. Furthermore, toxicity tests of JZG have executed in SD rats, with 6 months of consecutively administration of JZG with dosages 16, 34, and 68 g/kg (equivalent to 20, 40, and 80 times that human dosage), respectively, no toxic response was observed (unpublished data) during the test and one month after. Therefore, all these researches support the clinical application of JZG in treating NAFL. In 2008, JZG got the permission and certificate from Chinese SFDA (no. 2008L11181) to perform the 2nd phase of clinical trial.

This is a randomed, double-blinded, and placebo-controlled clinical trial to assess the effect of TCM medication in NAFL patients. In this trial, JZG was found to significantly reduce hepatic steatosis in NAFL patients compared to placebo control; major improvements were demonstrated through CT evaluation, with the change of L/S ratio. In addition, BMI was also markedly decreased after 24-week JZG intervention compared with the baseline.

Although our findings indicated that JZG was superior to placebo in some outcomes, the tentatively positive effects shown in the present study should be interpreted with caution. There are a number of limitations in this study. Firstly, the diagnosis of NAFL was through CT evaluation and lack of the histology evidence. Secondly, to be convenient for the patients, we used standard dosing of JZG according to the latest Chinese Medicine Recipe Dictionary (1997); thus, the dosage was not so exact when it was applied to people with different bodyweights and heights. Thirdly, the lifestyle deviations among patients may have influence on the results more or less, and some factors are uncontrollable.

## 5. Conclusion

We have found in our study that JZG is superior to placebo in increasing L/S ratio of CT evaluation and reducing BMI. This randomized, placebo-controlled, and double-blind clinical trial further consolidated evidence for the safety and effectiveness of JZG in treating patients with NAFL.

## Figures and Tables

**Table 1:** Baseline characteristics of patients (FAS analysis).

Parameter	JZG (*n* = 111)	Placebo (*n* = 110)	*P*
Age, y	42.39 ± 11.55	44.82 ± 11.41	0.1178
Male sex, *n* (%)	94 (84.68)	83 (75.45)	0.0858
Weight (male), kg	78.87 ± 10.38	79.43 ± 10.70	0.7280
Weight (female), kg	63.22 ± 7.32	64.49 ± 9.50	0.5334
Systolic pressure, mmHg/L	124.44 ± 8.98	122.21 ± 9.40	0.0724
Diastolic pressure, mmHg/L	77.69 ± 6.52	77.18 ± 6.96	0.5732
L/S ratio	0.74 ± 0.21	0.79 ± 0.18	0.0856
BMI	26.85 ± 3.34	26.69 ± 2.78	0.6994
Course of NAFL, month	26.61 ± 29.29	30.02 ± 34.59	0.6004
Comorbidity (with), *n* (%)	4 (3.60)	4 (3.64)	1.0000
History of drug hypersensitivity (yes), *n* (%)	2 (1.80)	1 (0.91)	n.s.
Combined medicine using (yes), *n* (%)	2 (1.82)	3 (2.73)	n.s.
Serum glucose, mmol/L	5.54 ± 0.37	5.27 ± 0.64	1.0000
ALT, IU/L	41.22 ± 19.48	42.61 ± 20.85	>0.05
AST, IU/L	32.99 ± 12.76	32.92 ± 13.31	>0.05
BUN, mmol/L	5.59 ± 6.76	8.91 ± 3.90	>0.05
Serum Cr, *μ*mol/L	79.84 ± 20.19	77.82 ± 17.24	>0.05

Data were presented as mean ± standard deviation.

**Table:** (a)

Follow-ups	FAS			PPS		
	JZG	Placebo	P*	JZG	Placebo	*P*
Baseline	0.74 ± 0.21	0.79 ± 0.18	0.0856	0.74 ± 0.21	0.78 ± 0.18	0.1366
Week 12	0.87 ± 0.22	0.82 ± 0.23		0.87 ± 0.22	0.82 ± 0.24	
Week 12-baseline	0.13 ± 0.18	0.03 ± 0.17	0.0003	0.13 ± 0.18	0.04 ± 0.18	0.0015
Week 24	0.99 ± 0.24	0.85 ± 0.27		1.00 ± 0.23	0.85 ± 0.28	
Week 24-baseline	0.25 ± 0.23	0.06 ± 0.21	0.0011	0.26 ± 0.23	0.07 ± 0.22	0.0003

*P values derived from paired *t*-tests for baselines comparison between 2 groups and Wilcoxon signed-rank tests for difference value of L/S ratio from baseline to week 12 and week 24 of treatment.

**Table:** (b)

	FAS		PPS	
	*F*	*P*	*F*	*P*
Group	38.85	<0.0001	36.19	<0.0001
Baseline	13.69	0.0003	11.35	0.0009
Center	7.25	0.0076	10.15	0.0017
Placebo group* (95% CI)	0.07 (0.03, 0.11)		0.07 (0.03, 0.12)	
JZG group* (95% CI)	0.24 (0.20, 0.28)		0.25 (0.21, 0.30)	
Mean change between JZG and placebo group (95% CI)	−0.18 (−0.24, −0.12)		−0.18 (−0.24, −0.12)	

*The values were for mean change in placebo group and mean change in JZG group of the complete treatment, respectively.

**Table 3:** The frequency of adverse effects/events (SS, *n* (%)).

Items	Placebo (*N* = 110)	JZG (*N* = 111)
ALT increase	9 (8.18)	4 (3.60)
AST increase	2 (1.82)	0 (0.00)
Serum TG increase	0 (0.00)	2 (1.80)
Sloppy stool	1 (0.91)	1 (0.90)
Exhaustion/dizziness	0 (0.00)	1 (0.90)
Dull pain in belly	1 (0.91)	0 (0.00)
Diarrhea	1 (0.91)	0 (0.00)
Cold	1 (0.91)	2 (1.80)
Cough	0 (0.00)	1 (0.90)
Expectoration	1 (0.91)	0 (0.00)
Yellow urine	1 (0.91)	3 (2.70)
Flush face	1 (0.91)	0 (0.00)
Sense of hunger	1 (0.91)	0 (0.00)
Injury	0 (0.00)	1 (0.90)
Stomach illness	0 (0.00)	1 (0.90)
Abnormal EKG	0 (0.00)	1 (0.90)
WBC increase	0 (0.00)	1 (0.90)
Dry throat	0 (0.00)	1 (0.90)

Total	19 (17.27)	19 (17.12)

**Table 4:** Adverse effects/events (SS analysis).

	Placebo (*n* = 110)			JZG (*n* = 111)			P*
	Patients	Cases	Incidence (%)	Patients	Cases	Incidence (%)	
Total AEs	14	19	12.73	16	19	14.41	0.7149
AEs related to study	0	0	0.00	2	2	1.80	0.4977

*P was calculated using CMH-*χ* ^2^ test for presence of any side effect.

## References

[1] Angulo P (2002). Medical progress: nonalcoholic fatty liver disease. *The New England Journal of Medicine*.

[2] Ruhl CE, Everhart JE (2004). Epidemiology of nonalcoholic fatty liver. *Clinics in Liver Disease*.

[3] Nugent C, Younossi ZM (2007). Evaluation and management of obesity-related nonalcoholic fatty liver disease. *Nature Clinical Practice Gastroenterology and Hepatology*.

[4] Marchesini G, Brizi M, Blanchi G (2001). Nonalcoholic fatty liver disease: a feature of the metabolic syndrome. *Diabetes*.

[5] Malaguarnera M, Di Rosa M, Nicoletti F, Malaguarnera L (2009). Molecular mechanisms involved in NAFLD progression. *Journal of Molecular Medicine*.

[6] Loria P, Adinolfi LE, Bellentani S (2010). Practice guidelines for the diagnosis and management of nonalcoholic fatty liver disease. A decalogue from the Italian Association for the Study of the Liver (AISF) Expert Committee. *Digestive and Liver Disease*.

[7] Méndez-Sánchez N, Arrese M, Zamora-Valdés D, Uribe M (2007). Treating nonalcoholic fatty liver disease. *Liver International*.

[8] Wang M, Sun S, Wu T (2013). Inhibition of LXRalpha/SREBP-1c-mediated hepatic steatosis by Jiang-Zhi granule. *Evidence-Based Complementary and Alternative Medicine*.

[9] Yang L-L, Wang M, Liu T (2011). Effects of Chinese herbal medicine Jiangzhi granule on expressions of liver X receptor a and sterol regulatory element-binding protein-1c in a rat model of non-alcoholic fatty liver disease. *Journal of Chinese Integrative Medicine*.

[10] Song HY, Zhang L, Pan JL, Yang LL, Ji G (2013). Bioactivity of five components of Chinese herbal formula Jiangzhi granules against hepatocellular steatosis. *Journal of Integrative Medicine*.

[11] Zeng MD, Fan JG, Lu LG (2008). Guidelines for the diagnosis and treatment of nonalcoholic fatty liver diseases. *Journal of Digestive Diseases*.

[12] Moher D, Schulz KF, Altman DG (2001). The CONSORT statement: revised recommendations for improving the quality of reports of parallel-group randomized trials. *Journal of the American Podiatric Medical Association*.

[13] Longo R, Ricci C, Masutti F (1993). Quantification by 1H localized magnetic resonance spectroscopy and comparison with computed tomography. *Investigative Radiology*.

[14] Ataseven H, Yildirim MH, Yalniz M (2003). Correlation between calibrated computerized tomographic findings and histopathologic grade/stage in non-alcoholic steatohepatitis. *Journal of Hepatology*.

[15] Bellentani S, Marino M (2009). Epidemiology and natural history of non-alcoholic fatty liver disease (NAFLD). *Annals of Hepatology*.

[16] Lu Y-L, Wang M, Zhang L (2010). Simultaneous determination of six components in the ’Jiang-Zhi’ granule by UPLC-MS analysis. *Chinese Journal of Natural Medicines*.

[17] Miao W, Tao L, Hua-feng W, Lian-jun X, Peng-yong Z, Guang J (2010). Clinical study of Jiangzhi Granule and behavioral intervention for nonalcoholic fatty liver disease of phlegm and blood-stasis syndrome. *Shanghai Journal of Traditional Chinese Medicine*.

[18] Zhang L, Zheng P, Ji G (2011). Jiangzhi granula ameliorate hepatic steatosis via improving leptin resistance in high—fat diet induced NAFLD rats. *Hepatology International*.

